# Coarse Particulate Air Pollution Associated with Increased Risk of Hospital Admissions for Respiratory Diseases in a Tropical City, Kaohsiung, Taiwan

**DOI:** 10.3390/ijerph121013053

**Published:** 2015-10-16

**Authors:** Meng-Hsuan Cheng, Hui-Fen Chiu, Chun-Yuh Yang

**Affiliations:** 1 Division of Pulmonary and Critical Medicine, Department of Internal Medicine, Kaohsiung Medical University Hospital, Kaohsiung 807, Taiwan; E-Mail: cmhkmu@gmail.com; 2 Graduate Institute of Medicine, Kaohsiung Medical University, Kaohsiung 807, Taiwan; 3 Department of Pharmacology, College of Medicine, Kaohsiung Medical University, Kaohsiung 807, Taiwan; E-Mail: chiu358@yahoo.com.tw; 4 Department of Public Health, College of Health Sciences, Kaohsiung Medical University, Kaohsiung 807, Taiwan; 5 Division of Environmental Health and Occupational Medicine, National Health Research Institute, Miaol 350, Taiwan

**Keywords:** fine particles, coarse particles, air pollution, case-crossover, respiratory diseases, hospital admissions

## Abstract

This study was undertaken to determine whether there was an association between coarse particles (PM_2.5–10_) levels and frequency of hospital admissions for respiratory diseases (RD) in Kaohsiung, Taiwan. Hospital admissions for RD including chronic obstructive pulmonary disease (COPD), asthma, and pneumonia, and ambient air pollution data levels for Kaohsiung were obtained for the period from 2006 to 2010. The relative risk of hospital admissions for RD was estimated using a case-crossover approach, controlling for weather variables, day of the week, seasonality, and long-term time trends. For the single pollutant model (without adjustment for other pollutants), increased rate of admissions for RD were significantly associated with higher coarse PM levels only on cool days (<25 °C), with a 10 µg/m^3^ elevation in PM_2.5–10_ concentrations associated with a 3% (95% CI = 1%–5%) rise in COPD admissions, 4% (95% CI = 1%–7%) increase in asthma admissions, and 3% (95% CI = 2%–4%) rise in pneumonia admissions. No significant associations were found between coarse particle levels and the number of hospital admissions for RD on warm days. In the two-pollutant models, PM_2.5–10_ levels remained significantly correlated with higher rate of RD admissions even controlling for sulfur dioxide, nitrogen dioxide, carbon monoxide, or ozone on cool days. This study provides evidence that higher levels of PM_2.5–10_ enhance the risk of hospital admissions for RD on cool days.

## 1. Introduction

Over the past decade, many epidemiologic studies demonstrated positive associations between ambient levels of particulate matter (PM) (generally measured as PM with an aerodynamic diameter < 10µm (PM_10_)) and daily mortality and hospital admissions or emergency room (ER) visits for cardiovascular and respiratory morbidity [1,2,3,4,5]. PM_10_ was seen as the most health relevant PM size fraction and therefore measured on a regular basis worldwide.

While previous studies primarily used PM_10_ as an exposure indicator, fine particles (defined as PM with an aerodynamic diameter less than 2.5 µm; PM_2.5_) have become a greater health and regulatory concern due to data based upon epidemiologic studies suggesting that PM_2.5_ might exert greater toxicity than larger particles [6,7,8,9,10]. It is now generally accepted that PM_2.5_ are more harmful to health than larger particles (PM_10_) because PM_2.5_ offer a greater surface area and hence potentially larger concentrations of adsorbed or condensed toxic air pollutants per unit mass [11,12,13]. Most of the prior studies only used PM_2.5_ or PM_10_ as particle measurements, leaving the effects of other particle sizes—particularly PM_2.5–10_—not well understood [6,14].

Fewer studies have examined the potential adverse health effects attributed to the coarse fraction, that is, particles ranging in size from 2.5µm to 10µm in aerodiameter (PM_2.5–10_) [14]. PM_2.5–10_ originate mainly from abrasive mechanical processes such as mechanical grinding, windblown dust, and agricultural activities. PM_2.5–10_ are predominantly composed of crustal-related materials such as calcium (Ca), magnesium (Mg), aluminum (Al), silicon, and iron (Fe), and primary organic materials such as pollen, spores, as well as plant and animal debris [11,15,16]. In contrast, the origin of chemical composition of PM_2.5_ is combustion-related constituents. PM_2.5_ are composed of many organic and inorganic compounds, including sulfate, nitrate, organic carbon and elemental carbon, carbonates, metals, and water [17,18]. The adverse health effects associated with ambient exposure to PM_2.5–10_ may thus differ from those of PM_2.5_ considering differences in the sites of deposition in the respiratory tract and sources and chemical composition for these two different-sized fractions [19].

Although the association between PM_2.5_ levels and adverse respiratory health outcomes (RD) is well established [5,12,20,21,22,23,24,25,26,27,28,29,30,31,32], epidemiologic data on the influence of PM_2.5–10_ on RD morbidity are limited and inconsistent [19,21,24,33,34,35,36,37,38,39,40,41]. More comprehensive knowledge of the health risks associated with exposure to PM_2.5–10_ is still needed as suggested in a review on this topic by Brunekreef and Forsberg (2005) [14], in which the authors concluded that coarse PM has a stronger or as strong a short-term effect on respiratory health as fine PM. Because above mentioned studies were conducted primarily in America and some European cities, the findings may not be applicable to Taiwan, which has different air pollutant mixtures and environmental conditions. 

This study was undertaken to examine the short-term associations of daily concentrations of PM_2.5–10_ with frequency of hospital admissions for RD including chronic obstructive pulmonary disease (COPD), asthma, and pneumonia among individuals residing in Kaohsiung city, a large industrial city in southwest Taiwan, over a 5 year period from 2006 to 2010, using a case-crossover design.

## 2. Materials and Methods

### 2.1. Kaohsiung City 

Kaohsiung is the largest commercial harbor and the second largest city in Taiwan, with a population of approximately 1.46 million. It is the chief center of the heavy industry, including China Steel Corporation and China Shipbuilding Corporation, and of the petrochemical industry situated on the southwest coast of Taiwan. The city is considered a symbol of recent success in Taiwan’s economic development.

### 2.2. Hospital Admission Data

The National Health Insurance (NHI) Program, which provides compulsory universal health insurance, was implemented in Taiwan on 1 March 1995. Most medical institutions (93%) are contracted to the Bureau of NHI (BNHI), and those not contracted provide fewer health services. More than 96% of the population who are covered by NHI used health services at least one time through contracted medical institutions. 

Computerized records of daily clinic visits or hospital admissions are available for each contracted medical institution. All medical institutions must submit standard claim documents for medical expenses on a computerized form, which includes the date of admission and discharge, identification number, gender, birthday, and diagnostic code of each admission. Therefore, the information from the NHI database appears to be sufficiently complete, reliable, and accurate for use in epidemiological studies. In total, 15,283,304 admissions were collected for the period of 2006–2010. Of the 15,283,304 admissions, 1,229,455 were recorded in Kaohsiung city (8.04% of all the admissions). Daily counts of hospital admissions with a primary diagnosis of RD were extracted from the medical insurance files for the period 2006–2010. RD outcomes included the following conditions: COPD (International Classification of Diseases, 9th revision (ICD-9) codes 490–492, 494, and 496), asthma (code 493), and pneumonia (codes 480–486). In addition, this study was approved by the ethics review board of Kaohsiung Medical University Hospital (KMUH-IRB-EXEMPT-20130018).

### 2.3. Pollutant and Meteorological Data 

**Figure 1:**
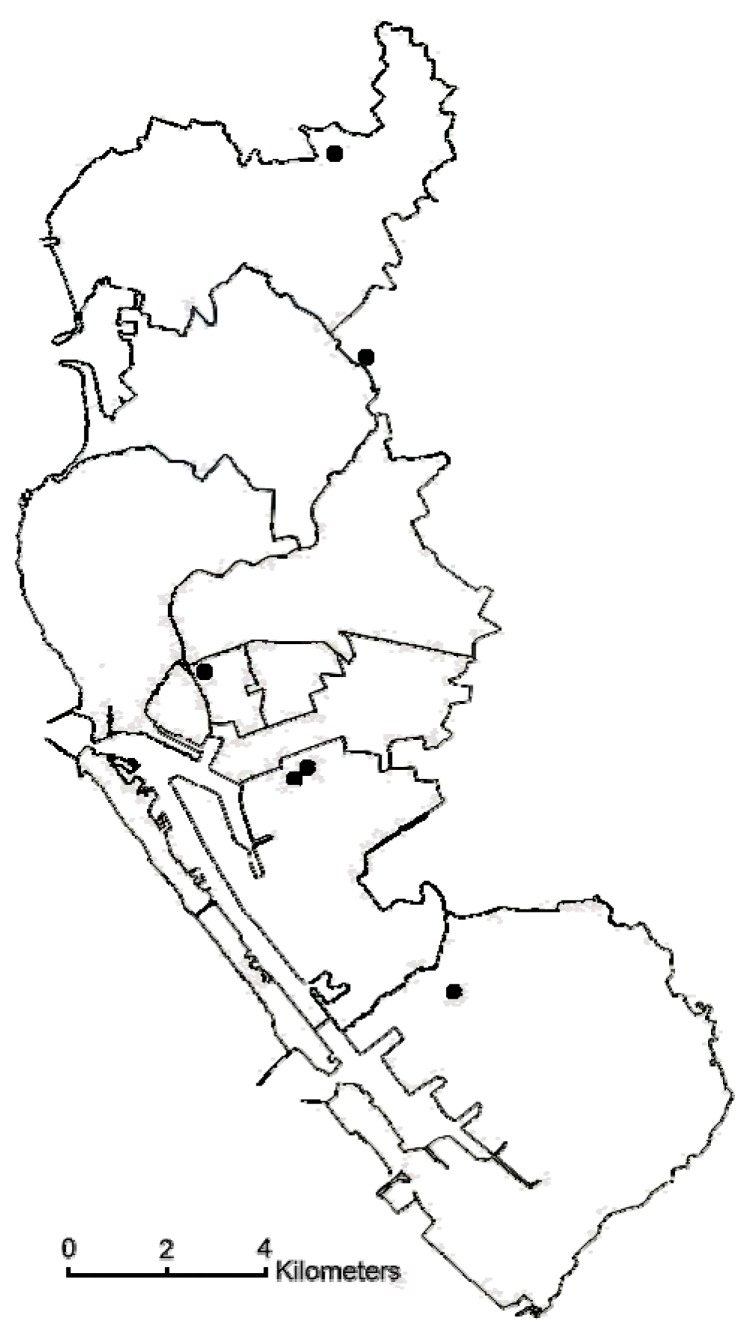
Map of Kaohsiung city showing location of the air quality monitoring stations.

Six air-quality monitoring stations were established in Kaohsiung city by the Taiwanese Environmental Protection Administration (EPA), a central governmental agency in 1994 (Figure 1). The stations used commercial monitoring instruments designated by the USEPA as an equivalent or reference method and manufactured by US Thermo Environmental Instruments, Inc. (Franklin, MA, USA). The monitoring stations were fully automated and routinely monitored 5 “criteria” pollutants including sulfur dioxide (SO_2_) (by ultraviolet fluorescence); particulate matter (PM_10_) (by beta-ray absorption); nitrogen dioxide (NO_2_) (by ultraviolet fluorescence), carbon monoxide (CO) (by nondispersive infrared photometry), and ozone (O_3_) (by ultraviolet photometry) levels. However, PM_2.5_ was not regularly monitored. PM_2.5_ concentrations in Taiwan were measured continuously since 2006. PM_2.5_ was measured using tapered element oscillating microbalance method samplers. The availability of the monitoring network for PM_2.5_ and the continuation of PM_10_ monitoring provided an opportunity to calculate coarse PM concentrations. The concentration of the coarse fraction was calculated by subtracting PM_2.5_ levels from PM_10_ levels. For each day, hourly air pollution data were obtained for all of the monitoring stations. After calculating the hourly mean of each pollutant from the 6 stations, the 24-hr average levels of these pollutants were computed. Daily information on mean temperature and mean humidity was provided by the Central Weather Bureau from a station located on the coastline of Kaohsiung Harbor.

### 2.4. Statistics 

Data were analyzed using the case-crossover technique [42,43,44]. This design is an alternative to Poisson time series regression models for studying the short-term effects attributed to air pollutants (Levy *et al.*, 2001). In general, the case-crossover design and the time-series approach yielded almost identical results [45,46].

The time-stratified approach was used for the case-crossover analysis [47]. A stratification of time into separate months was made to select referent days as the days falling on the same day of the week within the same month as the index day. Air pollution levels during the case period were compared with exposures occurring on all referent days. This time-stratified referent selection scheme minimizes bias due to non-stationarity of air pollution time-series data. [48,49,50] Results of previous investigations indicated that increased number of hospital admissions for RD were associated with higher ambient air pollutant levels on the same day or the previous two days [51]. Longer lag times have rarely been described. Thus the cumulative lag period up to 2 previous days (*i.e.*, the average air pollutant levels of the same and previous 2 days) was used. Because pollutants vary considerably by season, especially O_3_ and PM, seasonal interactions between PM and hospital admissions have often been reported. However, previous studies were conducted mostly in countries where the climates are substantially different from that in Kaohsiung [52,53], which has a tropical climate with no apparent 4-season cycle. Hence in this study the potential interactions of seasonality on the adverse effects of PM were not considered, but temperature was used instead. The adverse health effects of each air pollutant were examined for the “warm” days (days with a mean temperature above 25 °C) and “cool” days (days with a mean temperature below 25 °C) separately. 

The associations between hospital admissions for RD and levels of PM were estimated using the odds ratio (OR) and their 95% confidence intervals (CI), which were generated using conditional logistic regression with weights equal to the number of hospital admissions on that day. All statistical analyses were performed using the SAS package (version 9.2; SAS Institute, Inc., Cary, NC). Both single-pollutant models and multi-pollutant models were fitted with a different combination of pollutants (up to two pollutants per model) to assess the stability of the effects of PM. Exposure levels to air pollutants were entered into the models as continuous variables. Meteorological variables such as daily average temperature and humidity on the same day, which might play a confounding role, were included in the model. Inclusion of barometric pressure did not markedly change the effect estimates and therefore was not considered in the final model. ORs were calculated on the basis of an increment in exposure corresponding to the 10 µg/m^3^ of PM_2.5_, PM_10_, and PM_2.5–10_. The criterion for significance was set at *p* < 0.05.

## 3. Results and Discussion

During the 5 years of the study, there were a total of 81,836 hospital admissions for RD (14,136 for COPD, 5222 for asthma, and 62,478 for pneumonia) for the 63 hospitals in Kaohsiung City. The descriptive statistics for hospital admissions and corresponding environmental data are shown in Table 1. The average fine and coarse PM during the study period was 45.88 µg/m^3^ and 31.65 µg/m^3^, respectively. Approximately 60% of PM_10_ mass may be attributed to PM_2.5_ mass. There was a mean of 44.82 daily hospital admissions for RD (7.74 for COPD, 2.86 for asthma, and 34.22 for pneumonia) in the city over the study period. 

Pearson’s correlation coefficients among the air pollutants are presented in Table 2. 

Table 3 shows the effect estimates of PM on frequency of hospital admissions for RD in single-pollutant and two-pollutant models. Data demonstrated that the relationship between PM and RD-related hospital admissions differed by season. Generally, no significant associations between PM and number of hospital admissions for RD were observed on warm days.

On cool days, there were consistent significant positive associations with RD-related hospital admissions in single pollutant model (without adjustment for other pollutants) for all PM (PM_2.5_, PM_10_, and PM_2.5–10_), with a 10 µg/m^3^ increase in PM_2.5_ associated with a 11% (95% CI = 9%–13%), 10% (95% CI = 6%–13%), and 12% (95% CI = 11%–13%) rise in COPD, asthma, and pneumonia admissions, respectively. A 10 µg/m^3^ elevation in PM_2.5__–10_ was associated with a 3% (95% CI = 1%–5%) increase in rate of COPD, 4% (95% CI = 1%–7%) asthma, and 3% (95% CI = 2%–4%) increase in pneumonia admissions. For PM_10_, the corresponding estimates were 5% (95% CI = 3%–6%), 4% (95% CI = 3%–6%), and 5% (95% CI = 4%–5%), respectively. PM_2.5_ showed more potent effect on RD-related admissions than PM_2.5__–10_ and PM_10_. In two-pollutant models, the influence of all PM on RD-related admissions generally remained significant after inclusion of other pollutants including SO_2_, NO_2_, CO, or O_3_ in the regression model. Similarly, PM_2.5_ demonstrated the greatest effect on rate of RD-related admissions. Compared to effect estimate associated with a 10 µg/m^3^ increase in PM_2.5_ levels, effect estimates of number of RD-related admissions associated with the same rise in PM_2.5__–10_ and PM_10_ levels were weaker. However, CI of respective effect estimates for PM_2.5__–10_ and PM_10_ overlapped. 

This study is one of the few that investigated the association between exposure to fine and coarse particles and rate of hospital admissions for RD. Data showed that the levels of fine and coarse particles were correlated with increased risk of hospital admissions for RD including COPD, asthma, and pneumonia, and only on cool days. In addition, PM_2.5_ demonstrated a stronger effect on RD-related admissions than PM_2.5–10_. 

The correlation between fine and coarse PM was moderate at values of 0.28–0.69 in previous studies with a higher value at 0.69 in Steubenville, USA [54]. In contrast, the relationship between PM_10_ and fine as well as coarse PM was greater [14]. The implication is that analyses based on PM_10_ are generally not able to support arguments on the relative importance of fine and coarse PM in the induction of RD. The correlation between fine and coarse PM (r = 0.64) in this study enables separation of the two effects but it was not possible to disentangle the joint effects of coarse and fine PM in a two-pollutant model given their correlation levels. It is unfortunate that thus far, few studies have reported the results from two-pollutant analyses.

Epidemiologic studies on the effects of coarse particles on hospitalizations for RD are limited and inconsistent. A study conducted in 6 French cities, Host *et al.* [21] found positive association between coarse PM and hospitalizations for respiratory infection, with an excess risk of 4.4% (95% CI = 0.9%–8.0%) per 10 µg/m^3^ increase in PM_2.5__–10_ levels. Qiu *et al*. [33] conducted a study in Hong Kong, and reported an interquartile range (IQR = 10.9µg/m^3^) increase of PM_2.5__–10_ was associated with 1.05% (95% CI = 0.19%–1.91%), 1.78% (0.41%–3.16%), and 0.27% (−2.42% to 3.03%) increase in ER visits for total RD, COPD, and asthma, respectively. A study in Hong Kong by Qiu *et al*. [34] reported a 3.33% (95% CI = 1.54%–5.15%) increase in ER visits for pneumonia per every 10µg/m^3^ increment of PM_2.5__–10_ in the past 4 days (lag 3). A study in 35 California counties by Malig *et al.* [35] reported a 10µg/m^3^ increment in the level of PM_2.5–10_ was associated with a 3.3% (95% CI = 2.0%–4.6%) increase in ER visits for asthma. In a large U.S. study of 108 counties, a 10µg/m^3^ increase in coarse PM was associated with a non-statistically significant unadjusted 0.33% (95% CI = −0.21% to 0.86%) increase in RD admissions and with a 0.26% (95% CI = −0.32% to 0.84%) increase in RD admissions when adjusted for PM_2.5_ on the same day [19]. A study conducted in 8 Southern European cities within the MED-PARTICLES projects, Stafoggia *et al.* [41] noted an increase of 1.24% (95% CI = −0.2% to 2.82%) in frequency of RD admissions for an IQR increase (6.3 µg/m^3^) in coarse PM levels at the cumulative lag_0–5_. In a Toronto, Canada study, Burnett *et al.* [37] reported a 4.8% (95% CI = 2.1%–7.6%) increase in the risk of RD admissions per 10 µg/m^3^ increase in PM_2.5–10_ level. However, significant associations observed for RD admissions, COPD, and respiratory infections in another Toronto study, were not present in gas-adjusted models [38]. In a study conducted in Vancouver, Chen *et al*. [36] reported a 1.03 (95% CI = 0.98–1.09) increase for the first RD admission, 1.22 (95% CI = 1.01–1.36) for the second RD admission, and 1.06 (95% CI = 1.02–1.11) for overall RD admissions for an increment of 4.2 µg/m^3^ in PM_2.5–10_ concentrations. Two studies conducted in the United Kingdom found no evidence of an association between RD admissions and exposure to PM_2.5__–10_ [39,40]. A study in Helsinki by Halonen *et al*. [24] reported no evidence of positive associations for pneumonia and asthma-COPD with coarse PM. In this study, a 10 µg/m^3^ increase in PM_2.5__–10_ in the 3-day moving mean concentrations on cool days was associated with a 3% (95% CI = 1%–5%) elevation in number of COPD admissions, 4% (95% CI = 1%–7%) in asthma admissions, and 3% (95% CI = 2%–4%) in pneumonia admissions. Corresponding relationships with a 10 µg/m^3^ increase in coarse PM in this study were higher than previously observed [33,34,35]. In addition, the association with PM_2.5_ concentration is almost three times larger than for coarse PM concentration in the two-pollutant model. This finding is consistent with the investigations that also found fine fraction to be correlated with greater risk per unit mass than the coarse fraction [19,24,41]. However, because coarse PM is obtained as the difference between PM_10_ and PM_2.5_, it is affected by measurement error from two sources, and the direction and magnitude of the resulting bias in the associations with hospitalizations cannot be predicted [41].

Only in recent years have researchers begun to separately address the adverse health effects attributed to PM_2.5–10_, because (1) PM_2.5–10_ were initially considered as potentially less toxic than PM_2.5_ due to their large size and small surface area to mass ratio and (2) it is only recently that PM_2.5_ were measured separately [21]. However, PM_2.5–10_ may plausibly impact health given their deposition in the lungs, high biological content such as organic matter and microbes, and, in urban areas, high content of heavy metals such as Fe and Al [55]. Further, particle agglomerates that are large enough to be in the coarse fraction may contain many ultrafine particles and other attached constituents [21]. 

Results from toxicological studies showed that, PM_2.5–10_ and PM_2.5_ both produce inflammatory effects, including some evidence that PM_2.5–10_ may be more inflammatory than PM_2.5_ [56,57,58,59,60,61]. Controlled exposure to concentrated ambient coarse particles can also produce an increase in neutrophils in healthy humans [62,63]. Such inflammatory responses are a major component of asthmatic disease and may incite or exacerbate other respiratory conditions [64,65]. 

Major PM_2.5–10_ components vary by region and by season, but typically include Ca, Mg, Al, silicon, and Fe, and primary organic materials such as pollen, spores, and plant and animal debris [11,15,16]. Despite considerable research, the relative toxicity of different constituents of PM_2.5–10_ remain unclear but likely vary dependent upon the components [60]. The origin of chemical pollutants in an urban atmosphere is known to be predominantly attributed to road traffic [3]. Concentrations in urban environments are generally more influenced by transportation than in rural conditions, in which agriculture, other sources such as unpaved and construction sites, and wind are key influences [11].

In our study, adverse effects were observed only on cool days. It was possible to confirm that observed PM-induced effects varied by season [8,66]. The observed seasonal variation in effect estimates might be explained by variation in exposure patterns. It might be related to the relatively mild winters in Kaohsiung, where the mean monthly temperatures from December to February ranged from 18.8 to 22.1 °C and the mean daily temperature rarely fell below 15 °C. People in Kaohsiung are therefore more likely to go outdoors and open the windows in the cool than warm season (higher exposure). Thus, monitored concentrations of coarse PM may be closer to personal exposure to PM_2.5–10_ in the cool than warm season (more reliable exposure assessment). This fact may attenuate the PM_2.5–10_ induced influence in the warm season. On the other hand, seasonal differences in air pollution mixtures may also modify the effect estimates. Air pollution mixtures may include a greater proportion of toxic components during cooler months. Further, compared with other studies in developed countries, our data demonstrated larger effect estimates per unit increase of PM_2.5–10_. One potential explanation for this discrepancy is that most published investigations only noted straightforward, pooled effect estimates (lack of effect estimates stratified by season). This may mask inherent differences between different climates and air pollution mixtures. Variations in seasonal and regional effect estimates may in part result from differences in the chemical composition of particles [66,67]. Nevertheless, the seasonal pattern of air pollution adverse health effects needs to be further investigated.

The case-crossover study design was proposed by Maclure [42] to examine the influence of transient, intermittent exposures on the subsequent risk of rare acute-onset events in close temporal proximity to exposure. This design offers the ability to control for many confounders by design rather than by statistical modelling. This design is an adaptation of the case-control study in which each case serves as his or her own referent. Therefore, time-invariant subject-specific variables such as gender, age, underlying chronic disease, or other individual-level characteristics do not act as confounders. In addition, the time-stratified approach was found to be effective in controlling for seasonality [47], time trends, chronic, and slowly varying potential confounders [49,50,68]. 

For a factor to confound the relationship between coarse PM levels and admissions for RD it needs to be correlated with both variables. It is unlikely that time-variant subject-specific variables such as smoking, diet, and other indoor pollutants confounded the present association since these factors either do not vary significantly over time or vary slowly. In addition, there is no reason to believe that daily variations in the individual risk factors are correlated with changes in PM_2.5–10_ air pollution [69]. 

As in other studies in this field, we derived daily average levels of PM_2.5–10_ from outdoor monitoring stations to represent the personal exposure to PM_2.5–10_. Numerous factors, such as air conditioning and ventilation rate between indoor and outdoor air, may affect the monitoring results from fixed stations as surrogates of personal exposure to PM_2.5–10_. PM_2.5–10_ levels typically are more spatially heterogeneous than PM_2.5_ due to its higher deposition velocities in the atmosphere for these higher mass particles [19,55]. Concentrations of PM have also been shown to vary across space based on proximity to different sources, making exposure assignment especially difficult given the limited numbers of monitoring stations with data to estimate PM_2.5–10_ [55]. The potential for exposure measurement error in epidemiologic studies based on monitoring stations is likely to be greater for investigating associations of health indicators with PM_2.5–10_ than PM_2.5_ [19]. Larger measurement error relative to PM_2.5_ may be a plausible explanation for weakened associations for PM_2.5–10_ in this study. Measurements of PM_2.5–10_ levels are indirect, estimated through subtraction of PM_2.5_ from PM_10_ concentrations measured at the same monitoring station in this study. While past investigations deemed this a reliable approach to estimating PM_2.5–10_ in urban areas [55], there are inherently errors due to the uncertainty of both filters. PM_2.5–10_ were assigned from fixed, outdoor monitoring stations to individuals to estimate exposure (assuming that exposure was homogeneous encompassing the entire studied area). Exposure measurement errors resulting from differences between the population average exposure and ambient PM_2.5–10_ levels are unavoidable. However, this type of error may underestimate the true effect of exposure-response association due to the non-differential misclassification of the exposure [51,70].

This study was conducted in a tropical city. These facts may somewhat restrict the generalizability of these findings to other locations with different meteorological and racial characteristics. Further, behavior such as air conditioning usage or time spent outdoors may affect personal exposures. This might modify the magnitude of the observed associations compared with other geographical locations.

**Table 1:** Distribution of daily respiratory diseases admissions, weather conditions, and air pollution variables in Kaohsiung, Taiwan, 2006–2010.

Variable ^a^	Min	Percentile			Max	Mean
		25%	50%	75%		
PM_10_ (μg/m^3^)	19.97	45.21	72.72	104.24	607.14	77.53
PM_2.5_ (μg/m^3^)	11.44	25.57	44.30	61.88	144.37	45.88
PM_2.5–10_(μg/m^3^)	4.95	17.63	27.87	42.07	489.56	31.65
SO_2_ (ppb)	1.91	6.02	7.49	9.20	22.45	7.80
NO_2_ (ppb)	4.45	15.45	21.69	28.81	53.11	22.31
CO (ppm)	0.13	0.41	0.56	0.71	1.34	0.57
O_3_ (ppb)	4.77	18.90	27.92	37.19	72.96	29.14
Temperature(°C)	12.88	22.65	26.81	29.08	32.21	25.68
Humidity (%)	40.35	68.15	72.30	75.94	93.55	72.10
Hospital admissions						
COPD	0	5	7	10	25	7.74
Asthma	0	1	3	4	12	2.86
Pneumonia	0	27	34	40	73	34.22

Abbreviation: Min, minimum value; Max, maximum value. ^a^ 24-hr average.

**Table 2:** Correlation coefficients among air pollutants.

Variable	PM_2.5_	PM_2.5–10_	SO_2_	NO_2_	CO	O_3_
**PM_10_**	0.92 *	0.89 *	0.25 *	0.74 *	0.72 *	0.37 *
**PM_2.5_**	-	0.64 *	0.25 *	0.80 *	0.81 *	0.42 *
**PM_2.5–10_**	-	-	0.19	0.53 *	0.47 *	0.24 *
**SO_2_**	-	-	1.0	0.40 *	0.40 *	−0.02
**NO_2_**	-	-	-	1.0	0.93 *	0.11
**CO**	-	-	-	-	1.0	0.14
**O_3_**	-	-	-	-	-	1.0

* *p* < 0.05.

**Table 3:** Percent increase (95% CI) of hospital admissions for respiratory diseases associated with a 10 μg/m^3^ increase of particulate matter in Kaohsiung, Taiwan, 2006–2010.

Temperature	PM_2.5_	PM_2.5-10_	PM_10_
≥ 25 °C (1125 days)			
COPD			
Without adjustment ^a^	1.00 (0.98–1.03)	1.02 (1.00–1.03)	1.01 (1.00–1.02)
Adjusted for SO_2_	1.00 (0.98–1.02)	1.02 (1.00–1.03)	1.01 (1.00–1.02)
Adjusted for NO_2_	0.98 (0.95–1.00)	1.01 (1.00–1.03)	1.00 (0.99–1.01)
Adjusted for CO	0.96 (0.93–0.99)	1.01 (1.00–1.03)	1.00 (0.99–1.01)
Adjusted for O_3_	0.93 (0.90–0.96)	1.01 (0.99–1.03)	0.99 (0.98–1.00)
Asthma			
Without adjustment ^a^	1.02 (0.98–1.06)	1.02 (1.00–1.05)	1.02 (1.00–1.04)
Adjusted for SO_2_	1.02 (0.98–1.06)	1.02 (0.99–1.05)	1.01 (1.00–1.03)
Adjusted for NO_2_	0.99 (0.95–1.04)	1.02 (0.99–1.04)	1.01 (0.99–1.03)
Adjusted for CO	0.97 (0.93–1.02)	1.02 (0.99–1.04)	1.00 (0.98–1.03)
Adjusted for O_3_	0.95 (0.91–1.00)	1.01 (0.99–1.04)	1.00 (0.98–1.02)
Pneumonia			
Without adjustment ^a^	1.00 (0.99–1.01)	1.02 (1.01–1.03)	1.01 (1.01–1.02)
Adjusted for SO_2_	1.00 (0.99–1.01)	1.02 (1.01–1.03)	1.01 (1.01–1.02)
Adjusted for NO_2_	0.97 (0.96–0.99)	1.02 (1.01–1.02)	1.00 (1.00–1.01)
Adjusted for CO	0.95 (0.94–0.97)	1.02 (1.01–1.02)	1.00 (0.99–1.01)
Adjusted for O_3_	0.93 (0.92–0.94)	1.01 (1.01–1.02)	0.99 (0.99–1.00)
< 25 °C (701 days)			
COPD			
Without adjustment ^a^	1.11 (1.09–1.13)	1.03 (1.01–1.05)	1.05 (1.03–1.06)
Adjusted for SO_2_	1.13 (1.11–1.16)	1.03 (1.02–1.05)	1.05 (1.04–1.06)
Adjusted for NO_2_	1.06 (1.03–1.09)	1.02 (1.00–1.04)	1.02 (1.01–1.04)
Adjusted for CO	1.05 (1.02–1.08)	1.02 (1.00–1.03)	1.02 (1.01–1.03)
Adjusted for O_3_	1.09 (1.07–1.12)	1.03 (1.01–1.04)	1.04 (1.03–1.05)
Asthma			
Without adjustment ^a^	1.10 (1.06–1.13)	1.04 (1.01–1.07)	1.04 (1.03–1.06)
Adjusted for SO_2_	1.12 (1.08–1.16)	1.04 (1.01–1.07)	1.05 (1.03–1.07)
Adjusted for NO_2_	1.05 (1.00–1.09)	1.02 (0.99–1.05)	1.02 (1.00–1.04)
Adjusted for CO	1.04 (0.99–1.09)	1.02 (0.99–1.05)	1.02 (1.00–1.04)
Adjusted for O_3_	1.08 (1.05–1.12)	1.03 (1.00–1.06)	1.04 (1.02–1.06)
Pneumonia			
Without adjustment ^a^	1.12 (1.11–1.13)	1.03 (1.02–1.04)	1.05 (1.04–1.05)
Adjusted for SO_2_	1.14 (1.13–1.15)	1.03 (1.03–1.04)	1.05 (1.05–1.06)
Adjusted for NO_2_	1.07 (1.06–1.08)	1.02 (1.01–1.02)	1.02 (1.02–1.03)
Adjusted for CO	1.06 (1.05–1.08)	1.01 (1.01–1.02)	1.02 (1.01–1.03)
Adjusted for O_3_	1.10 (1.09–1.11)	1.03 (1.02–1.03)	1.04 (1.03–1.04)

^a^ single pollutant model.

## 4. Conclusions 

In summary, this study provided evidence of associations between short-term exposure to coarse PM and increased risk of rate of hospital admissions for RD. Associations were much more pronounced during the cool period. These findings reinforce the possible role of PM_2.5–10_ on the number of hospital admissions for RD.
